# News

**DOI:** 10.1155/S1463924699000097

**Published:** 1999


Enso Italia S.r.1., Piazza S. Camillo de Lellis, 1, I-20124 Milan. Tel: +39 2 670 871; Fax: +39 2 669 7516.

Fins Verkoopkantoor/Enso (Holland) BV, Postbus 12386, 1100 AJ Amsterdam. Tel: 020-6505555; Fax: 020-6505622.

Enso International, 281 Tresser Blvd., 15th Floor, Stamford, CT 06901. Tel: +1 203 978 1755; Fax: +1 203 978 1237.

Enso International, 2550 Leavenworth St., Suite 2, San Francisco, CA 94133. Tel: +1 415 567 1295; Fax: +1 415 567 1281.

Enso Interamericas, 8750 Doral Boulevard, Suite 270, Miami, FL 33178. Tel: +1 305 716 9799; Fax: +1 305 716 5441.

T.S.P., 2 Main Street, Chatham, New Jersey 07928. Tel: +1 201 635 3530; Fax: +1 201 635 1680.

Paper Agencies (Aust.) Pty. Ltd., 554 Burwood Road, Hawthorn, Vic. 3122. Tel: +61 3 9819 4911; Fax: +61 3 9819 4250.

Paper Agencies (Aust.) Pty. Ltd., Suite 5, 102 Alfred Street, Milsons Point, NSW 2061. Tel: +61 2 9957 3177 Fax: +61 2 9957 1505.

Enso France S.A., 15/25 Boulevard Amiral Bruix, F-75782 Paris Cedex 16. Tel: 01 53 64 79 00; Fax: 01 53 64 79 90.

S.A. Comptoir Finlandais N.V., Avenue des Gaulois 7, B-1040 Bruxelles. Tel: 02 743.13.60 Fax: 02 736.01.78.

Enso (Schweiz) AG, Bahnhofstrasse 13, CH-8808 Pfäffikon SZ. Tel: 055 415 3060; Fax: 055 415 3069. (Egalement pour le Liechtenstein)

Enso (Deutschland) GmbH, Große Bleichen 30, D-20354 Hamburg. Tel: 040 350 990; Fax: 040 3509 9146.

Enso Española S.A., Apartado 76 – 08760 Martorell, Barcelona. Tel: 93 631 1000; Fax: 93 631 1021.

Enso Sverige AB, Joakim Stockhaus, försäljningsdirektör, Box 2017, SE-750 02 Uppsala, Sweden. Tel: 018 65 30 00; Fax: 018 65 30 10. E-mail: oakim.stockhaus@up.mkt.enso.com

### News

### Solutia reviewing options for phosphorus derivatives business

*St. Louis, 1 July 1998:* Solutia (NYSE:SOI) said today that it is reviewing options for its phosphorus derivatives business, including sale, alliance and joint venture, and has retained the investment banking firm Goldman Sachs to assist in the evaluation.

"The phosphorus derivatives business has been profitable for Solutia," said Michael E. Miller, Solutia vice chairman, "but it's clear that global consolidation in some form in this industry is likely to happen. To fulfill management's commitment to the Board of Directors and to our stockholders, it's imperative that we explore value-enhancing opportunities for our businesses".

### News from the September issue of Performance chemicals international

*Surfactants*: It is a time of change in the surfactants sector. Increasingly, demanding customer requirements in both the industrial and personal care areas, as well as the growing importance of environmental considerations, are encouraging change right across the sector. Mirroring the trend in the rest of the industry, M&A activity has been intense over the past year or so as we move towards fewer but larger players. The sector is consolidating in an attempt to improve its profitability, a phenomenon seen throughout the speciality sector which is making life harder for the smaller player.

*Food additives*: The market for food additives is characterized by increasing sophistication with demand mainly propelled by the rapidly growing processed foods sector. As lifestyles change and food processing technology evolves, there is increasing demand for a variety of food additives, ranging from sweeteners to thickeners. 'Consumer preference in foods and flavours is continually changing due to fashion, lifestyle changes and the focus on healthy eating,' says Manoj Gujral, business director and general manager at Oxford Chemicals, a company that specializes in the manufacture and supply of speciality aroma chemicals to the global market for flavours and fragrances.

*Ireland*: Ireland is becoming a key location for the European pharmaceutical and chemical industry. More than 120 overseas companies employ 15 000 people in this sector in Ireland. In 1997, pharmaceutical products worth more than $7bn were exported. This represents 15% of total exports and makes Ireland one of the world's largest exporters of pharmaceuticals and fine chemicals—sectors that dominate the Irish chemical industry. The heavy and petrochemical industry is not well represented due to a lack of raw materials and the capital-intensive nature of this end of the industry.

*Catalysts*: Catalysts remain one of the more innovative areas of chemistry with new technologies being developed that could have dramatic impacts on he economics of chemical processes worldwide. In the USA, a researcher has suggested a method of using combinatorial chemistry to discover new solid-state catalysts. In a paper published in the latest edition of *Nature* magazine, Selim Sankan of the chemical engineering department at the University of

California, Los Angeles, suggests a 'mass screening' technique that could speed up the current trial and error process. The pharamaceutical industry already uses combinatorial synthesis methods, in combination with mass-screening techniques, to speed up the discovery of new drugs.

*Technical alert*: A US consortium has announced what it describes as a 'a major milestone' in the development of biotechnological routes to chemicals from renewable resources. The consortium members are Genencor International, Eastman Chemical, Electrosynthesis Company, MicroGenomics and Argonne National Laboratory, a Department of Energy (DOE) lab. The first target chemical for the process is ascorbic acid, which has a world market worth $600m. The consortium says the process could cut the capital cost by half compared to the conventional Reichstein process.

For more information, call the Editor Alan Tyler at 44-181-652 8126, email alantyler@rbi.co.uk.

- *Performance Chemicals International* is published by the Reed Chemical Group, part of Reed Business Information (RBI). The Reed Chemical Group also includes *Asia-Pacific Chemicals, Asian Chemical News, European Chemical News, Chemical Insight* and 24 h online service Chemical News and Intelligence (CNI).
- RBI publishes over 60 titles including: *New Scientist, Flight International, Estates Gazette, Contract Journal, Commercial Motor, Doctor, Bankers Almanac, Kelly's Business Directory, Kompass British Exports* and many more. For a full listing see RBI's web site http://www.reedbusiness.com

### Top five chemical companies generated $134.6 bn in sales in 1997, new analysis shows

The five largest chemical companies in the world generated sales of $134.6 billion in 1997 and profits before tax totalling $15.5 billion, according to *Chemical Insight*'s analysis of annual financial performance in the chemical industry. Combined net profits for the top five were $7.7 billion.

The 1997 analysis puts four US companies at the head of the performance league table—Union Carbide, Georgia Gulf, Rohm & Haas, and Nalco. These are followed by one of Europe's largest petrochemical producers, Borealis, and Britain's pharmaceuticals, agrochemicals and speciality chemicals producer, Zeneca. The chemical industry is heading for hard times, but the analysis for 1997 shows just how much stronger some of the European producers have become and their good standing—in 1997 at least—among their peers.

This critical comparison looks at the performance of companies across the globe. It assesses performance according to 32 financial measures and ratios, and looks at the industry in ways which compare year-on-year performance, profitability, productivity and financial health. The analysis is all about comparisons across national boundaries, regions and accounting conventions. It shows which companies performed well in 1997 and points to future strengths.

The analysis issue contains detailed financial information on the top 30 companies in the chemical industry. It ranks companies by sales and gives net profits and net margins, and research and development and capital spending figures. Productivity is an important element of the analysis, and the productivity data published rank companies by sales per employee and give figures for value added, employee numbers and employee costs.

The industry is facing difficult times now, and Union Carbide is not alone is issuing a third quarter 1998 profits warning. However, average industry sales rose by 7% last year. Cash flows were 9% higher and pre-tax profits up by 12%.

Chemical Insight *is published by Reed Chemical Publications in the UK, Reed Business Information (RBI), a division of Reed Elsevier. Other chemical publications published by RBI include* European Chemical News, Asia Pacific Chemicals, Performance Chemicals International, *and* Chemical News & Intelligence, *a worldwide, Internet-based 24 h news service. Reed Business Information also publishes over 60 titles, including:* Flight International, Estates Gazette, Contract Journal, Commercial Motor, Doctor, Bankers Almanac, Kelly's Business Directory, Kompass British Exports, *and many more. For a full listing see RBI's web site—http://www.reedbusiness.com. For further details about* Chemical Insight, *contact the Editor, Nigel Davis, e-mail: nigel.davis@rbi.co.uk, Tel: +44 (0)181 652 3397, Fax: +44 (0)181 652 8952*

### Patent application fees abolished

Small firms and private individuals benefitted most when the United Kingdom Patent Office abolished the application fee for patents from 1 October 1998. The Office is the first in the industrialized world not to charge a patent application fee.

Abolition of the filing fee forms part of a 20% cut in Patent Office fees that came into effect on 1 October. Other reductions include a cut in the trademark application fee from £225 to £200, a cut in trademark registration renewal from £250 to £200, and cuts in patent renewal fees by an average of 18%.

The cuts assist entry into the systems of patents, trademarks and registered designs, and encourage their use by small firms and private individuals. The greatest savings to be made are in patent renewals in the earlier years, when companies are frequently still in the phase of product development and have yet to make a return on their investment. Savings to industry will equal £12 million, or 20% of the Patent Office's fee income.

Welcoming the fee reductions, Patent Office chief executive Paul Hartnack said: 'The reductions in Patent Office fees make a significant contribution to the competitiveness of British industry, particularly among the small firms which are the source of innovation and creativity in Britain'.

'The fee reductions have been made possible by the continuous and successful efforts of management and staff to raise the quality of service and reduce unit costs since the Patent Office relocated to Newport, South Wales, in 1991'.

Details of the fee reductions are available on the Patent Office Web site at www.patent.gov.uk/snews/notices/red-fee.html. Copies of the statutory instruments are available from the Stationery Office Bookshops and from the Patent Office. They are *The Trade Marks (Fees) Rules 1998* (SI 1998 No 1776), £1.10; *The Registered Designs (Fees) Rules 1998* (SI 1998 No 1777), £1.10 and *The Patents (Fees) Rules 1998* (SI 1998 No 1778), £1.95).

*For information, contact Brian Caswell, The Patent Office, +44 (0)1633 814729 or Michael Binns, Prowse & Co, +44 (0)1372 363386.*

### 21st century requirements to monitor our environment prompts action by The Royal Society of Chemistry

Modern instrumentation has allowed us to push back the frontiers of detection such that we are able to determine incredibly small amounts of natural and anthropogenic pollutants and contaminants in our environment, whether they are in our homes, workplaces, cities, the countryside or the oceans. The fact that we can detect these pollutants in minuscule amounts does not necessarily mean that the levels present in the environment are harmful to our health or well being, but it does drive world-wide legislation on these substances. Therefore, there is a requirement to monitor, ascertain the sources, prevent the release, develop better detection methods and make properly assessed scientific judgements on the toxicity, exposure and risk assessment of the pollutants to which we are exposed in our daily lives.

The Royal Society of Chemistry has recognized the importance of these 21st century requirements, and that it is essential to promote and disseminate the knowledge of newly developed technologies for monitoring our various environments. Therefore, it is launching the *Journal of Environmental Monitoring (JEM)* which is dedicated to assessing exposure and health risks through the latest developments in measurement science. The journal, with the first issue due to be published in February 1999 and then bi-monthly thereafter, is unique in that it aims to publish all the relevant information on this subject area in one source.

This journal is intended for environmental and health professionals in industry, and officials from governmental and regulatory agencies as well as research scientists interested in the environment.

"I think the journal will be of interest to all analytical scientists involved with environmental monitoring issues. Currently at NIOSH, environmental monitoring is one of the key components of the National Occupational Research Agenda (NORA)". Dr E. R. Kennedy, NIOSH, Cincinnati, USA.

"Environmental contaminants are becoming the No. 1 public concern". Mr J. V. Dutton, Consultant, UK.

"The launching of a journal dedicated to environmental monitoring with some emphasis on legislative issues is an excellent idea". Dr. Philippe Quevauviller, European Commission, DGXII SM&T Programme, Belgium.

The Royal Society of Chemistry (RSC) is a learned society with a worldwide membership of 46 000. it has as its main objectives the advancement of the science of chemistry and its applications, and the maintenance of high standards of competence and integrity among practising chemists. The RSC markets a comprehensive range of high quality information products and services.

For further details contact: Harpal Minhas, The Royal Society of Chemistry, Thomas Graham House, Science Park, Milton Road, Cambridge CN4 4WF, UK. Tel: +44 (0)1223 432292; Fax: +44 (0)1223 420247; e-mail: jem@rsc.org; URL: www.rsc.org/jem

### Meetings reports

### Micromass host international seminar on the enabling role of MS

Micromass, the mass spectrometry people, hosted *MS in Proteomics & Drug Discovery: An International Seminar on the Enabling Role of MS.* The seminar, which was open to the public and incorporated Micromass' 1998 organic MS users' meeting, the first event of its kind to be organized by the company since its re-formation in 1996.

The seminar saw a move from central Manchester, the traditional home of the Micromass user meeting, to Shrigley Hall in Cheshire. From 18th to 20th October 1998, this impressive 19th century country mansion housed an international panel of invited speakers and Micromass users, presenting an insight into emerging MS-based strategies in proteomics and contemporary drug discovery/development. Speakers included Roland Annan (SmithKline Beecham, PA, USA), Walter Blackstock (Glaxo Wellcome, UK), Daryl Pappin (Imperial Cancer Research Foundation, UK) and Jeffrey Kiplinger (Pfizer, Groton, CT, USA).

Originally planned as a 'stand-alone' meeting to be hosted, earlier in 1998, by Glaxo-Wellcome, the proteomics sessions were, through unexpectedly popular demand, rescheduled at this larger venue. Protein identification using MS and bio-informatics is becoming

